# Fighting cervical cancer in Africa: Taking a closer look at human papillomavirus 35

**DOI:** 10.4102/ajlm.v13i1.2243

**Published:** 2024-01-29

**Authors:** Sophia U. Okeke

**Affiliations:** 1 Department of Medicine and Surgery, Faculty of Clinical Sciences, College of Medicine, University of Ibadan, Ibadan, Nigeria; 2 College Research and Innovation Hub, University of Ibadan, Ibadan, Nigeria

## Introduction

Recent findings show that global cancer mortality has fallen by 32% in the past 24 years.^1^ Although this is good progress, it becomes evident, when examined in a geographical context, that Africa still lags behind. While the continent has seen improvements in diagnostics, there is still much to be desired in aspects of cancer prevention and treatment. Although 57% of all new cancer cases are recorded in low- and middle-income countries,^1^ cancer research output from these areas, including sub-Saharan Africa, remains low. Furthermore, it is apparent that there are significant differences in the types of cancers constituting a burden across different regions of the world. While cervical cancer accounts for 3.3% of cancer-related deaths worldwide,^2^ 24.55% of these deaths occur in sub-Saharan Africa.^3^

## Human papillomavirus 35 in women of African ancestry

The steep decline in cervical cancer rates in countries with a high development index may be attributed to the implementation of suitable nationwide vaccination and screening programmes.^4^ Just as there is a difference in cervical cancer burden, there is also a difference in the prevalence of high-risk human papillomavirus (HPV) strains. Although HPV 16 and 18 account for 70% of HPV-positive cervical cancer cases,^5^ various epidemiological studies have shown that another high-risk HPV genotype, HPV 35, which is not covered by the available vaccines,^6^ accounts for a significant number of positive cases in sub-Saharan Africa.^6,7,8^ The largest HPV epidemiological study, conducted by the Clinical Genetics Branch of the National Cancer Institute, United States, further established the strong association between HPV 35 and precancerous lesions in women of African ancestry.^9^ This study also reported a 10-fold increase in the prevalence of cervical intraepithelial neoplasia-3 among women of African ancestry infected with HPV 35 compared to white women.^9^ They also showed that certain single-nucleotide polymorphisms in the E7 oncogene were peculiar to African-American women.^9^ In contrast to other regions, HPV 35 is associated with between 4% and 10% of invasive cervical cancer cases in sub-Saharan African women.

Various studies done across subpopulations in Africa have reported high prevalence rates of HPV 35 in women with cervical intraepithelial neoplasia-3. Mbulawa and colleagues, working in South Africa in 2022, reported HPV 35 as the second-most dominant genotype detected in women with cervical intraepithelial neoplasia-3 (22.2%), and the fourth-most in those with cervical cancer (12.5%).^6^ A larger study that investigated cervical biopsy samples of 659 women from three African countries (Ghana, Nigeria, and South Africa) also identified HPV 35 in 9.7% of cervical biopsies, making it the fourth-most prevalent HPV genotype in that cohort.^7^

Other studies have observed a relationship between HPV 35 infections, HIV, and high-grade cervical intraepithelial lesions and have shown an increased variability in the L1 capsid (a major component of the current HPV vaccines) of HPV 35 in women living with HIV.^8^ Another study conducted amongst Zimbabwean women found a 13-base-pair insertion in the Long Control Region of the HPV 35 genome relative to the reference HPV 35 genome from the United States.^10^ However, associations between these genetic variations and cervical dysplasia are yet to be described. Human papillomavirus 35 has also been found to be the second-most prevalent and persistent HPV infection in HIV-positive women.^11^ A systematic review conducted in 2020 revealed that, amongst women in Ethiopia, there was a marked difference in the prevalence of high-risk HPV strains depending on HIV status, with HPV 35 accounting for 10% of infections in HIV-positive individuals.^12^ Interestingly, a systematic review in sub-Saharan Africa by Okoye et al. in 2021 regarding prevalent HPV genotypes in HIV patients showed that HIV-positive women with HPV 35 were twice as likely to develop cervical cancer compared to those with HPV 16 infections.^13^

## Urgent need for studies on the genomic epidemiology of human papillomavirus 35 in African women

These marked disparities observed amongst HPV in African women and the peculiarity of HPV 35 cannot continue to be neglected, as they have major implications for funding and prioritisation of HPV and cervical cancer research. Despite its apparent association with a significant proportion of cervical dysplasia in African women, there is a paucity of studies on the genomics and biology of HPV 35. Why does HPV 35 have a stronger predilection for African women? Is there something in the genome of Africans that increases the tissue tropism or persistent infectivity of HPV 35? What could be the role of HIV in HPV 35 infections? To address these lingering questions, the comprehensive genomic epidemiology of HPV infection among African women needs to be described. This is important, as the approved HPV vaccines do not cover HPV 35.

Advancements in high-throughput technologies and next-generation sequencing allow not just DNA analysis but also analysis of genomic expression profiles in protein products, as well as the study of epigenomic patterns such as the heterogeneous methylation observed in high-risk HPV precancerous lesions.^14^ Although genomic advances continue to bring us closer to the goal of precision medicine, Africa is being left behind due to inadequate representation in the data being generated. Despite Africa having the most diverse population, only a small amount of genomic data from Africa is available for decision-making. To achieve global health equity in cervical cancer care and prevention, genomics-informed research with adequate representation of Africans is key. We must start generating genomic data in Africa to allow sufficient evidence to drive the implementation of inclusive cancer policies.

## Role of genomics in developing an improved multivalent vaccine that targets human papillomavirus 35

Genomic technologies allowed coronavirus disease 2019 scientists to make significant progress, by sequencing the genomes of coronavirus disease 2019 strains and translating that knowledge to vaccine design. Although some studies have shown that available vaccines offer cross-protection to other oncogenic HPV types, care must be taken in the interpretation of these studies due to the importance of various underlying factors such as HIV status and the occurrence of multiple-type HPV infections.

A study conducted among participants in a Gardasil vaccine trial back in 2007 showed that the quantity of antibodies required to neutralise HPV 45 (not covered by the vaccine) was about 10 to 100 times greater than that for HPV 18, even though they share the same phylogenetic origin.^15^ We need to conduct more specific studies on the effectiveness of these vaccines against HPV 35 and identify the basis for cross-protection and impact. This would help the scientific community establish the exact efficacy of the available HPV vaccines for African women.

Reverse vaccinology, which was applied in the development of the meningococcal vaccine,^16^ could be used in developing better HPV vaccine candidates. A thorough analysis of various HPV 35 sublineages would allow the detection of novel HPV antigens and inform the rapid development of effective multi-epitope vaccines. Since host genetic factors may be responsible for the kind of HPV infections seen, studies should investigate candidate host genes involved in the immune response and genetic variations that may negatively impact HPV vaccination strategies. To further refine the available vaccines, the design and implementation of genome-wide association studies using data obtained across Africa could help to identify certain high-risk loci in the human genome that mediate increased susceptibility to HPV 35 infections and to identify the precise genomic relationships between HPV 35 and HIV infections.

## Need for collaborative efforts in upscaling human papillomavirus 35 research in Africa

As much as governments are saddled with providing basic services such as HPV vaccination and improving access to HPV screening to meet up with the global cervical cancer elimination target,^17^ we must always remember that research plays a major role in improving the effectiveness and quality of patient care. A recent study conducted of the genomes of 910 people of African descent showed that around 300 million base pairs of their genes and genomic regions were not present in the reference human genome.^18^ This poses a significant problem in the level of information we can harness about human diseases. Previous phylogenetic analysis revealed that HPV 16, which is a close relative of HPV 35, originated from Africa before dispersal into other populations.^19^ With the strong association of HPV 35 with cervical cancer in African women, there is a need for stakeholders to make efforts to drive research that would unearth the uniqueness and pathogenic mechanisms of HPV 35 among African women. Generating the necessary scientific evidence would provide a strong basis for the implementation of policies enabling the coverage of HPV 35 by the available vaccines.

Currently, there is a dearth of trained scientists, necessary equipment, and funding to tackle some of the fundamental research questions surrounding HPV 35 carcinogenicity. National government research agencies, private corporations, foreign research foundations, and academic institutions need to come together to promote HPV 35 research. A good example of collaborations that have driven genomics-based research on the African continent is the H3Africa consortium, an initiative of the United States’ National Institutes of Health, and Wellcome Sanger Institute that has empowered thousands of scientists from Gambia, Nigeria, Ethiopia, Uganda, and Botswana to produce quality research. One major discovery achieved through this scientific partnership was that, according to research on a study population from Nigeria and Ghana, Africans tend to have a special variant of the gene encoding apolipoprotein L1, thus resulting in an increased risk of kidney disease.^20^ The East Africa HPV Consortium has also conducted research on various aspects of HPV, including delineating the immunohistochemical differences in cervical intraepithelial neoplasia-2 and -3 between HIV-negative and HIV-positive patients.^21^ These efforts should be emulated across other regions of sub-Saharan Africa.

## Conclusion

The lessons learnt from the coronavirus disease 2019 and HIV pandemics have been logical, precise, and unambiguous – for sustainable progress to be made, African scientists must be empowered to solve challenges in Africa for Africa. Genomics studies are vital to elucidating the molecular epidemiology of HPV in Africa, understanding the associated risk factors and carcinogenic potential of endemic genotypes like HPV 35, and designing effective vaccines to capture the HPV diversity within the African population. Thus, there is an urgent need to improve genomics capacity in Africa given its potential role in reducing the comparatively high burden of cervical cancer on the continent. To achieve equity, we must ensure an adequate representation of data from Africa in the sequence data available to research scientists and industries. This can be achieved by funding genomic research in Africa, building capacity, and raising awareness of these issues within the scientific community.
